# A Toolbox and Crowdsourcing Platform for Automatic Labeling of Independent Components in Electroencephalography

**DOI:** 10.3389/fninf.2021.720229

**Published:** 2021-12-02

**Authors:** Gurgen Soghoyan, Alexander Ledovsky, Maxim Nekrashevich, Olga Martynova, Irina Polikanova, Galina Portnova, Anna Rebreikina, Olga Sysoeva, Maxim Sharaev

**Affiliations:** ^1^Center for Bioelectric Interfaces, National Research University Higher School of Economics, Moscow, Russia; ^2^Laboratory of Human Higher Nervous Activity, Institute of Higher Nervous Activity and Neurophysiology, Russian Academy of Sciences, Moscow, Russia; ^3^Research Center in AI, Skolkovo Institute of Science and Technology, Moscow, Russia; ^4^Faculty of Biology and Biotechnology, National Research University Higher School of Economics, Moscow, Russia

**Keywords:** EEG, automatic preprocessing, ICA, children, automatic artifact detection, machine learning algorithms

## Abstract

Independent Component Analysis (ICA) is a conventional approach to exclude non-brain signals such as eye movements and muscle artifacts from electroencephalography (EEG). A rejection of independent components (ICs) is usually performed in semiautomatic mode and requires experts’ involvement. As also revealed by our study, experts’ opinions about the nature of a component often disagree, highlighting the need to develop a robust and sustainable automatic system for EEG ICs classification. The current article presents a toolbox and crowdsourcing platform for Automatic Labeling of Independent Components in Electroencephalography (ALICE) available via link http://alice.adase.org/. The ALICE toolbox aims to build a sustainable algorithm to remove artifacts and find specific patterns in EEG signals using ICA decomposition based on accumulated experts’ knowledge. The difference from previous toolboxes is that the ALICE project will accumulate different benchmarks based on crowdsourced visual labeling of ICs collected from publicly available and in-house EEG recordings. The choice of labeling is based on the estimation of IC time-series, IC amplitude topography, and spectral power distribution. The platform allows supervised machine learning (ML) model training and re-training on available data subsamples for better performance in specific tasks (i.e., movement artifact detection in healthy or autistic children). Also, current research implements the novel strategy for consentient labeling of ICs by several experts. The provided baseline model could detect noisy IC and components related to the functional brain oscillations such as alpha and mu rhythm. The ALICE project implies the creation and constant replenishment of the IC database, which will improve ML algorithms for automatic labeling and extraction of non-brain signals from EEG. The toolbox and current dataset are open-source and freely available to the researcher community.

## Introduction

Electroencephalography (EEG) signal reflects the bioelectrical activity of brain neuronal networks. For more than a century, human neuroscience and clinical research applied scalp EEG recording to study and assess a broad scope of sensory and cognitive functions. One of the crucial steps of EEG preprocessing is “purifying” the brain signal by extraction of the electrical activity of non-neuronal origins such as eye movements and muscle artifacts. For recent decades, Independent Component Analysis (ICA) offered a solution to this problem based on the isolation of statistically independent sources called independent components (ICs) as linear combinations of signals from electrodes (Makeig et al., 1996; Delorme and Makeig, 2004). A source of each IC can be projected onto the electrode cap and estimated via timecourse and spectral power. For example, ICA allows identifying components related to eye-movement and muscle artifacts based on their bioelectrical signals’ specific characteristics, e.g., frequency and spatial distribution (Chaumon et al., 2015; Frølich et al., 2015). However, due to other frequent contaminations of EEG, a rejection of non-brain ICs is usually performed in the semiautomatic mode under the visual inspection of researchers. Herewith, labelings of ICs by different experts can substantially disagree, which might considerably affect the further analysis and reproducibility of EEG results (Robbins et al., 2020). Artifact rejection by ICA in children and patient EEG is especially challenging even for experts. The dependence of EEG analysis from subjective opinions of experts may explain that EEG data have been rarely included in large-scale studies or meta-analyses. For this reason, the automatic algorithms for EEG processing are the main objectives of many research groups (Nolan et al., 2010; Mognon et al., 2011; Winkler et al., 2011; da Cruz et al., 2018; Tamburro et al., 2018; Pedroni et al., 2019).

To create a robust and sustainable automatic system for EEG ICs classification, one needs to extract the most informative features from ICs and have an appropriate machine learning (ML) model inside the system. The accurate labeling of ICs is the crucial step in training and validating this model. The training of ML algorithms to automatically identify artifactual ICs will allow to set up a more objective methodology for EEG preprocessing.

Currently, a limited number of projects aims to create an automatic cleaning system of the EEG signal. For example, Automatic EEG artifact Detection based on the Joint Use of Spatial and Temporal features (ADJUST) (Mognon et al., 2011) and Fully Automated Statistical Thresholding for EEG artifact Rejection (FASTER) (Nolan et al., 2010) use empirical threshold-based algorithms. Machine learning approach was introduced in Multiple Artifact Rejection Algorithm (MARA) (Winkler et al., 2011), algorithms from the studies of Frølich et al. (2015) and Tamburro et al. (2018). SASICA software (Chaumon et al., 2015) is an EEGLAB Matlab plug-in (Chaumon et al., 2015), includes ADJUST, MARA, FASTER, and some other methods. The more novel study describes Adjusted-ADJUST approach (Leach et al., 2020) that is known as an advanced version for the previously described ADJUST software. It is aiming to produce automatic labeling for the pediatric ICA that differs from the ICA of adults because of infant EEG features. The suggested approach shows the higher quality even for adult data. All these studies used their private datasets for training and validation purposes. Those datasets were relatively small, consisting of several hundred ICs. In most cases, each IC was annotated by only one expert, which complicates the estimation of algorithm actual performance and comparison with other algorithms. Moreover, the lack of a large dataset with verified annotation limits the potential performance of machine learning models.

Pion-Tonachini et al. (2019) addressed this problem by proposing ICLabel Toolbox, which includes the annotation tool with crowdsourcing mechanics, datasets, and several machine learning algorithms. The annotation tool provides an interface to label a particular IC from the database by visualizing different components’ characteristics. In this toolbox, the ML algorithms are based on artificial neural networks and claimed to be the fastest and most accurate than other studies.

While the ICLabel project is an excellent resource for automatic artifact rejection in EEG, it has several drawbacks. The first one is potentially insufficient annotation quality as a non-expert user can annotate ICs. It means that even if an ML algorithm has high accuracy, the predicted classes may be wrong as ICs have no order or intrinsic interpretations and their classification by experts requires practice. Potential technical issues that prevent the best performance from experts are inability to see other ICs from the same EEG record, which is helpful in ambiguous cases (e.g., horizontal eyes component can consist of two ICs, so seeing them in parallel helps to infer their nature) and limitation of component time-window plots to only 3 s ranges. Clinical experts usually require at least 30 s to properly detect various slow-wave components or alpha rhythm, hardly detected in a short time interval. Another limitation of ICLabel that the authors themselves pointed to is a limited variety of EEG data (Pion-Tonachini et al., 2019), as their dataset does not contain data from infants and most clinical groups.

The current study presents a toolbox and crowdsourcing platform for Automatic Labeling of Independent Components in Electroencephalography (ALICE), which is available via link http://alice.adase.org/. The ALICE toolbox aims to build a sustainable algorithm to remove artifacts and find specific patterns in EEG signals using ICA decomposition. The presented toolbox was also designed to overcome the limitations of the previous approaches mentioned above.

For developing a sustainable ML-based EEG component classification, the proposed toolbox should have two components: a high-quality labeled dataset of ICs and a proper ML pipeline to train and validate models.

Thus, the first aim of the ALICE project was to create a high-quality dataset with IC labels. In order to achieve this goal, we performed the following steps:

• The definition of a rigorous set of possible IC classes that would cover a wide variety of cases and be easily understandable by experts.
• The annotation of IC reliability by combining opinions from multiple experts.
• Resolving the possible poor concordance between experts by various merging strategies.
• Attracting researchers to share their datasets, including unique EEG recordings from rare clinical groups.

The second aim of the ALICE project was to develop a robust but flexible ML pipeline for automated IC classification. The ML module includes implementing various features (both well-established and original), multiple ML models, and the validation pipeline. The ALICE project also invites the research community to develop their models using our dataset, which is available via link http://alice.adase.org/downloads.

The other ambitious goal for ALICE development is the automatic identification of components related to the functional brain oscillations, such as alpha and mu rhythm. Mu rhythm overlaps with alpha rhythm in a frequency range of 8-13 Hz but has a different oscillation shape and localization at scalp electrodes. While alpha rhythm is recorded predominantly from the occipital lobes with closed eyes and suppressed when eyes open (Berger, 1931), mu rhythm emerges over the sensorimotor cortex and is attenuated during movements. Importantly, mu rhythm does not react to opening or closing the eyes (Kuhlman, 1978). Despite the described differences, the automatic separation of mu from alpha waves in EEG is challenging and drawing the attention of many methodological studies (Cuellar and del Toro, 2017; Garakh et al., 2020). Still, the identification of mu rhythm often requires visual inspection and expertise. The ALICE toolbox aims to accumulate expert labeling of alpha and mu rhythms to improve automatic identification of functional brain oscillations by supervised ML.

## Materials and Methods

### Automatic Labeling of Independent Components in Electroencephalography Toolbox High-Level Architecture

**FIGURE 1:**
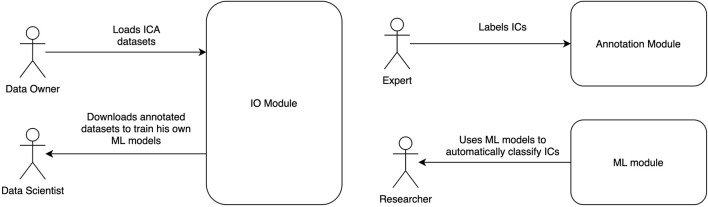
ALICE toolbox high-level architecture and user roles. Annotation module UI serves for Data Owners to upload IC data to the database and for Experts to provide annotation on existing or newly uploaded data. Data Scientists and Researchers work with ML module: the former train models based on selected samples from the database, the latter take pretrained models to work with their own data (online or offline). Online version of the ALICE toolbox is available at http://alice.adase.org/.

Automatic Labeling of Independent Components in Electroencephalography contains two modules (Figure 1):

• Annotation module, which consists of a user interface (UI) and ICs database. An HTTP API allows uploading ICs data to the database. Web-based UI allows experts to label uploaded data for future ML models training and validation.
• ML module is based on a Python library, which trains ML models based on expert annotations and uses pre-trained ML models to apply to new IC data.

### Annotation Module

By annotation, we mean a process of manual IC labeling by experts based on various data visualization tools available at the ALICE platform, such as IC topographic mapping, plots of time series, and power spectrum. An expert may choose IC labels from a predefined number of options.

We propose a set of IC component labels including major artifact types with subtypes as well as brain signal subtypes:

• Eye artifacts – eye movement artifacts of any type.
• Horizontal eye movements – components that represent activity during eye movements in horizontal directions.
• Vertical eye movements – components that represent activity during eye movements in vertical directions.
• Line noise – line current noise evoked by surrounding electrical devices.
• Channel noise – the noise associated with channels that can be Or.
• Brain activity – brain activity of any type.
• Alpha activity – alpha rhythm with oscillation in the frequency band of 8–13 Hz with predominance in the occipital lobe channels.
• Mu activity – mu rhythm with oscillation in the frequency band of 8–13 Hz with predominance or dipole localization in the frontal-central-parietal area.
• Muscle activity – artifacts from a recording of muscle activity on the head surface.
• Heartbeat artifacts – artifacts that represent electrocardiographic activity.
• Other – components with explicit nature that label is not listed in the labeling system, for example, breathing (experts could comment on the label choice in the comments section, the ALICE developers collect data from comments and expand the list of labels in the subsequent versions of the toolbox).
• Uncertain – components with unclear nature.

**FIGURE 2:**
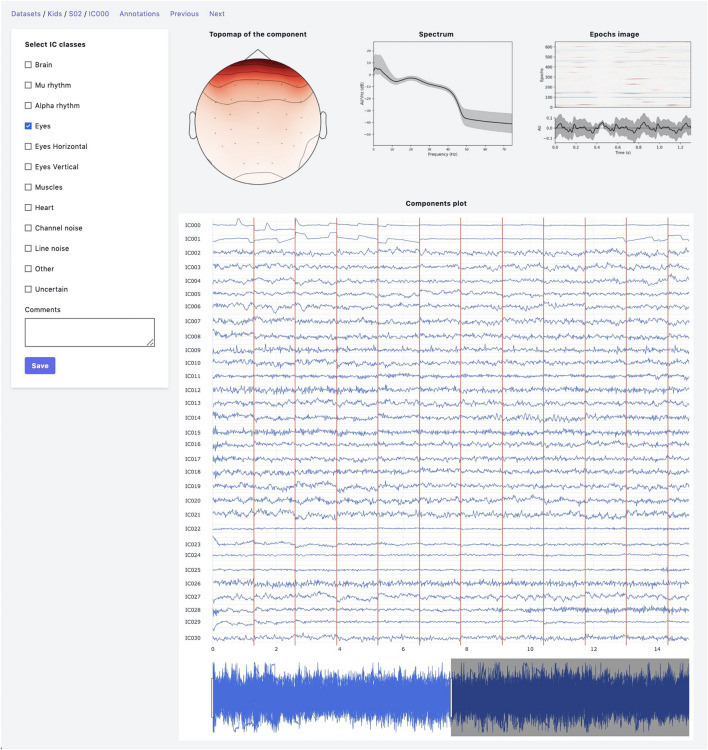
Annotation module interface. Top row: IC topomap and spectrum as well as Epochs image, illustrating the color-coded amplitude fluctuations (arbitrary units) of the IC in all available epochs (time relative to sound presentation on the *x*-axis, epochs on the *y*-axis). Image at the bottom shows averaged values of ICs timeseries; bottom row: all IC for considered subject are plotted together.

The web-based UI supports the annotation process (Figure 2). An expert has the following data visualization options:

• Topomap of IC weights.
• Power spectrum density plot.
• Plot of all ICs time series for the current subject (the time-series length is 30 s with the possibility of scrolling and zooming selected time interval).
• Epoch image illustrates the color-coded amplitude fluctuations of the IC in all available EEG epochs and averaged ICs time series values.
• This plot is helpful for the annotation of epoched data.

After a particular expert has finished the labeling process, the data of ICs with annotations can be packed into an archive by the annotation module by an administrator. Then, annotated data becomes available at the Downloads page of the ALICE toolbox and could be used both by the experts and ALICE data scientists.

### Machine Learning Pipeline

**FIGURE 3:**
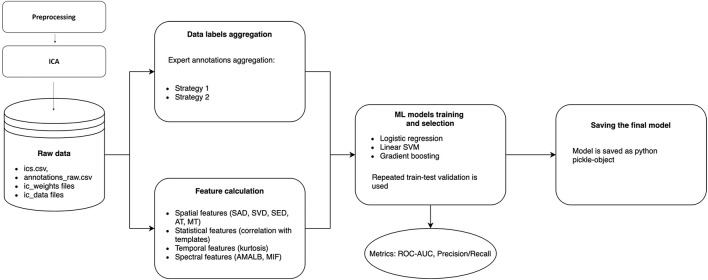
Data processing and machine learning pipeline in ALICE. Raw ICs data with annotations are passed to Data labels aggregation and Feature calculation blocks to form a labeled dataset and extract informative features from ICs. Three ML models are trained with repeated train-test validation and different model quality metrics are calculated. The best model is then selected and could be exported as a Python pickle-object.

There could be many discrepancies between experts’ annotations due to ambiguities in IC patterns, data quality, and differences at the expert level. The annotation inconsistency means that we need to create final IC labels in the dataset as a function of the individual annotations. So, before conventional ML pipeline steps, such as *Feature calculation* and *ML model training and selection*, we need to include an additional step – *Data label aggregation*. The whole data processing and ML pipeline are presented in Figure 3, and each step is discussed in detail below.

#### Data Labels Aggregation

**FIGURE 4:**
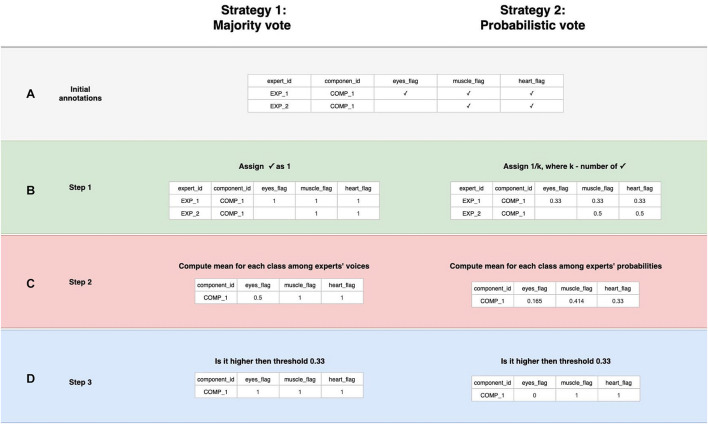
Data labeling strategies. There are several annotations for one component from various experts, but we strive to designate its belonging to a particular class strategies of data belling aggregation, those are “Majority vote” and “Probabilistic vote.” **(A)** Is a table of annotations of a specific component. **(B)** Transform into a matrix of *probabilities* that each component belongs to a particular class. **(C)** Group the experts’ opinions using the mean of the *probabilities* to obtain a *weighted probability*. **(D)** Determine whether the weighted probability is higher than the threshold or not.

This part aims to create a boolean variable between each component and each IC class, reflecting whether a specific activity is present or not in a particular component. The first step is to create an annotation table (Figure 4A). *The annotation* is a term denoting the labeling produced by an expert to a particular component. Experts have their own unique opinion about the component’s ICA class. Our goal is to develop an approach to grouping expert annotations to form a common opinion on each component.

A simple voting strategy seems to be a logically correct option: if most experts choose that a component contains a particular activity, for example, an eye artifact, then this component is classified as an eye artifact. This approach is the basis of Strategy 1, which we called *“Majority vote*,” although it does not require that the majority (more than 50%) of the experts assign the component this particular label. The threshold value can be changed. We provided an example where it equals to 33% which means we expect agreement over 33% of experts. In other words, by grouping experts’ annotations, we form the average of the experts’ votes (Figure 4C). We will consider this average value as the *probability* of assigning the component to a specific class. If the probability is higher than the threshold, we assume that the component encodes the given IC class; otherwise, it does not (Figure 4D).

**FIGURE 5:**
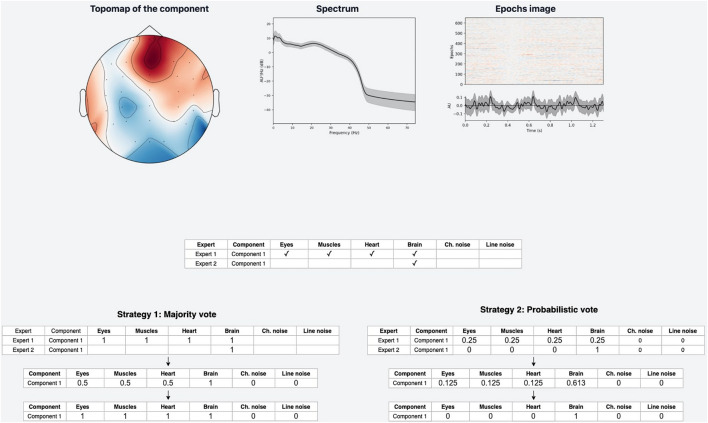
Discrepancies in experts’ opinions and difference in strategies handling. Experts may have different approaches in labeling samples with mixed nature of activity. Expert 1 marked such components with all activity types that are present in the component. Expert 2 instead focused on the components that have a clear pattern of a single IC class. Within a provided example we can see how the difference in their approaches lead to different annotations for the same IC component. The application of “Majority vote” and “Probabilistic vote” strategies for the suggested example affect significantly the final label of the component. With the first one the component corresponds to many IC classes, while in the second case the component is assigned only as a Brain activity.

Nevertheless, if an expert assigned a component to several classes, it means s/he recognizes several types of activity present in the IC. This situation can lead to ambiguous results if the expert acted with an approach where s/he labels mixed components with all types of activity s/he believes are potentially intermixed in a particular IC. If we were to use *Majority vote* for such situations, it would lead to low quality of the target variable as IC with only one label is a more genuine representation of this class than the component that contains a mixture of artificial and brain activity. An example of what this can affect is illustrated in Figure 5. We see that the component, due to such markup, is assigned to all classes simultaneously.

In order to overcome this situation, Strategy 2 was developed and titled “Probabilistic vote.” Imagine that, when labeling a component, an expert has one vote, which they equally distributed among all the classes to which they attributed this component. In other words, if a person marks a component as eyes and as muscles, and as heart, then with a *probability* of 0.33, they assign it to each of these classes (Figure 4B). Further, these *probabilities* are again averaged (Figure 4C). Then, a threshold is chosen, according to which it is decided whether this *weighted probability* will be transformed to 1 or 0 (Figure 4D). The threshold of 0.33 was chosen as the optimal threshold for the current data, assuming that components that consist of three or fewer labels still represent the simple pattern of interest for the model. This approach is rather valuable for cases where the mixed nature of components can affect the target variable; Figure 5 provides the example.

The threshold value is highly dependent on the level of agreement between experts since a too tight threshold with a low agreement will significantly reduce the number of objects. On the other hand, a weak threshold with a high agreement will lead to noisy, ambiguous components in the training set. We decided to use an equal threshold of 0.33 for both strategies. The threshold change for *Majority vote* will make sense with an increase in the number of experts.

#### Agreement Between Experts

We also computed metrics of expert agreement to be able to compare annotation quality of various classes as well as datasets. For the case of two experts, we propose using Cohen’s kappa (Cohen, 1960).


$$\kappa =\frac{p_{0}-p_{e}}{1-p_{e}}$$

Where *p*_*0*_ is the relative observed agreement (similar to accuracy), *p*_*e*_ is the hypothetical probability of agreement by chance.

For the case of multiple experts, we propose using Fleiss’ kappa (Fleiss and Cohen, 1973), which has a similar formula with a different definition of *p*_*0*_ and *p*_*e*_, that depend on weighted estimates. Basically, that shows the level agreement between the multiple experts above the value of agreement expected by chance for details refer to Fleiss and Cohen (1973).

Based on the metrics from Pion-Tonachini et al. (2019), we computed the inter-expert correlation between experts to compare our level of agreement with the level of agreement in ICLabel.


$$I\,E\,C=\frac{1}{N}\,\sum_{n=1}^{N}C\,o\,r\,r\,\left(v_{1,n},v_{2,n}\right)$$

*N*, number of components marked by both experts; *v*_*1,n*_, annotation vector made by the 1st expert corresponding to the *n*^*th*^ component; *v*_*2,n*_, annotation vector made by the 2nd expert corresponding to the *n*^*th*^ component.

All computational details about data label aggregation are available via link https://github.com/ledovsky/alice-eeg-ml that we share with interested researchers who might achieve higher performance rates on our dataset using their settings for strategies and thresholds.

#### Features Calculation

To reduce data dimensionality while preserving the most characteristic information for each IC class, we calculate specific temporal and spatial features of each signal. Most features are well established and based on previous research. Still, we introduced some modifications to existing ones and treated them as new features.

Among the established features are:

• Kurtosis of the component time series (Nolan et al., 2010; Mognon et al., 2011; Winkler et al., 2011; Tamburro et al., 2018). By definition, kurtosis is the fourth standardized moment of the time series. In epoched data, we calculate an average of the feature computed for each epoch separately. It helps to distinguish ICs that correspond to eyes and brain activity.
• Maximum Epoch Variance (Mognon et al., 2011; Tamburro et al., 2018) is used to detect eye movements. The value of this feature is a ratio between the maximum of the signal variance over epochs and the mean signal variance. As proposed in Mognon et al. (2011), we excluded one percent of the largest variance values to improve its robustness when calculating this feature.
• Spatial Average Difference (SAD), Spatial Variance Difference (SVD), and Spatial Eye Difference (SED). Spatial features proposed in Mognon et al. (2011) depend on IC weights of eyes-related electrodes. SAD is calculated as the difference between channel weight averages over frontal and posterior regions. SVD is the difference between weight variances in these regions. These are used to distinguish vertical eye movements. SED is the difference between the absolute values of weight averages in the left eye and right areas. This feature detects horizontal eye movements.
• Myogenic identification feature (MIF) (Tamburro et al., 2018) is used to detect muscle activity and is calculated as the relative strength of the signal in the 20–100 Hz band.
• Correlations with manually selected signal patterns (Tamburro et al., 2018). We use these to detect eye blinks and eye movements.

The ALICE toolbox also offers a possibility of mu and alpha rhythms annotation and classification. Thus, some features must be specific to these components’ spatial and temporal properties.

Alpha rhythm is known to be localized in occipital and parietal areas with increased power in 8–12 Hz for adults. Close to the alpha band frequency, mu rhythm is generated in central and frontal areas. We used those electrodes that maximally emphasize the contrast between mu and alpha localization by the topography-related features. Thus, the original features include:

• Mu topography (MT): A feature which is sensitive to topomaps of mu rhythm ICs, where *Mu* is the following set of electrodes in 10–20: “Fp1,” “Fpz,” “Fp2,” “F3,” “Fz,” “F4,” “Fc3,” “Fcz,” “Fc4,” “C3,” “Cz,” “C4.”

$$M\,T=\sum_{e\in M\,u}\left|w_{e}\right|-\sum_{e\notin M\,u}\left|w_{e}\right|$$

• Alpha topography (AT): A feature which is sensitive to topomaps of alpha rhythm ICs where *A* is the following set of electrodes in 10–20: “C3,” “Cz,” “C4,” “Cp3,” “Cpz,” “Cp4,” “P3,” “Pz,” “P4,” “O1,” “Oz,” “O2.”

$$A\,T=\sum_{e\in A}\left|w_{e}\right|-\sum_{e\notin A}\left|w_{e}\right|$$

• Average magnitude in alpha band (AMALB): The ratio between average amplitude in the alpha band (6–12 Hz) and average amplitude in other frequencies (0–6 Hz; 13–125 Hz) is sensitive to alpha ICs. The alpha range was expended to 6 Hz because alpha band tends to be in the lower frequency range for children (Marshall et al., 2002; Lyakso et al., 2020).

$$A\,M\,A\,L\,B=\frac{\sum_{f\in \left[6,12\right]}\underline{x}\,\left(f\right)}{\sum_{f\notin \left[6,12\right]}\underline{x}\,\left(f\right)}$$

Source code used to compute the features can be found via link https://github.com/ledovsky/alice-eeg-ml.

#### Machine Learning Models Training and Selection

The current version of ALICE Toolbox provides three different machine learning models: logistic regression (LR), linear support vector machine (SVM), and gradient boosting (XGB). These models are built on different principles and are relatively simple compared to neural networks and deep neural networks. Keeping in mind a relatively small initial dataset, we considered the three models mentioned above as an optimal initial model choice. All of them are optionally available for new training and testing procedures in ALICE. In particular, we used the LR implementation from scikit-learn package (Pedregosa et al., 2011) with default parameters (including regularization parameter C = 1.0, L2 penalty and liblinear solver). Linear SVM is taken from scikit-learn package (Pedregosa et al., 2011) with default parameters (including regularization parameter C = 1.0). Finally, we used the XGB model implementation from XGBoost package (Chen and Guestrin, 2016) with default patameters of 30 estimators with a maximal depth of 4.

In the ALICE, we implement the repeated train-test split cross-validation technique. We trained the model on 70% of samples and validated on the rest 30% with repeated train-test cross-validation and did not optimize any hyperparameters on cross-validation. We performed this procedure 50 times using different random train-test splits, estimating three main metrics of classification accuracy: Area Under the Receiver Operating Characteristic Curve (ROC-AUC), Area Under the Precision-Recall Curve (PR-AUC) and F1-score using the implementation of scikit-learn package (Pedregosa et al., 2011). ROC-AUC and PR-AUC were used as overall metrics of model performance for different thresholds and considered the main ones. F1 was used as a performance metric of optimal model splits and was considered as an additional metric.

Thorough code used for computations is open access https://github.com/ledovsky/alice-eeg-ml/blob/main/Basic%20Pipeline.ipynb. Thus, any person can go through our pipeline and make his/her changes to achieve higher results and easily compare them with our original performance rates. The Basic Pipeline explains how the models may be applied to any dataset.

### Initial Dataset

The ALICE project aims to involve the neurophysiological community in labeling existing publicly available and new IC datasets to improve ML models’ quality. However, the Baseline model trained on the dataset provided by IHNA&NPh RAS is already available to users.

Electroencephalography data were recorded using the NeuroTravel amplifier (EB Neuro, Italy) with sampling rate 500 Hz, and with 31-scalp electrodes arranged according to the international 10–10 system and included the following electrodes: “Fp1,” “Fpz,” “Fp2,” “F3,” “Fz,” “F4,” “F7,” “F8,” “FC3,” “FCz,” “FC4,” “FT7,” “FT8,” “C3,” “Cz,” “C4,” “CP3,” “CPz,” “CP4,” “P3,” “Pz,” “P4,” “TP8,” “TP7,” “T3,” “T4,” “T5,” “T6,” “O1,” “Oz,” “O2.” Ear lobe electrodes were used as reference, and the grounding electrode was placed centrally on the forehead. The initial dataset consists of recordings from 20 typically developing children aged 5–14 years. Within the experiment’s framework, sound stimulation was performed according to the odd-ball paradigm with a standard stimulus of 1,000 Hz and two deviant stimuli at 980 and 1,020 Hz. The interstimulus interval was 400 ms. Stimulus intensity were 75 dB.

Obtained data were filtered (0.1–40 Hz) and divided into epochs (−500; 800 s), where noisy epochs were removed by threshold (350 mV). Only the first 650 epochs of recording gained from the first 650 presentations of stimuli were used for posterior ICA decomposition (FASTICA) with resampling on the level of 250 Hz. Final data that were uploaded into ALICE consisted of 30 ICA components. All preprocessing steps were done using the MNE Python package (Gramfort, 2013).

The data annotation for training the Baseline model was carried out by two experts – experienced scientists of the Institute of Higher Nervous Activity and Neurophysiology of RAS. The first expert is a clinical neurologist, while the second one clinical psychologist; both experts had more that 15 years of experience in analysis of pediatric EEG ICA. For the correct work with the program, they received an instruction, which outlined the main steps they took when working with ALICE. Experts’ main task was set as follows – to mark each component using the set of labels: Eyes, Horizontal eye movements, Vertical eye movements, Line noise, Channel noise, Brain, Alpha activity, Mu activity, Muscle activity, Other, Uncertain. Following the instructions, if an expert saw that a component consisted of several activity types, s/he can assign the component to several classes. For example, among annotated components, there were often components marked both as eye artifacts and muscle activity simultaneously.

### Additional Datasets

For additional validation we used another dataset with children EEG. The recordings of 17 children aged 5–14 years were decomposed using ICA. Data were recorded using the same EEG system as in initial dataset. Participants watched series of videos in terms of the experimental paradigm. The collected data were filtered in the range 3–40 Hz, no other processing steps were applied. The same experts were asked to mark only those components that correspond to eye artifacts. The overall number of components was 149. The dataset is marked as Children dataset 2.

To test how ALICE performs on adult data the recordings of 21 adults were added to the ALICE platform. The experimental design, EEG system and data processing steps were the same as we used in the initial dataset. The data were annotated by four new experts. To facilitate the labeling process, the task for experts was to label only those components that correspond to eye artifacts. The datasets is called Adults’ dataset.

These additional datasets allowed us to estimate model performance when trained using initial dataset and re-trained on additional datasets. Moreover, it was mentioned previously that adult ICA and children ICA automatic labeling require different approaches to modeling. Thus, the second dataset allowed us to check whether suggested approach is suitable for EEG of any age, whereas the first dataset was acquired from the same cohort of participants. In order to assess model generalizability, data preprocessing was also different: the first dataset was prepared with different ICA method – AMICA (Palmer et al., 2011).

### Ethics Statement

The datasets were obtained from the research project (A physiological profile of autism spectrum disorders: a study of brain rhythms and auditory evoked potentials). It is conducted according to the guidelines of the Declaration of Helsinki and approved by the Ethics Committee of the Institute of Higher Nervous Activity and Neurophysiology (protocol code 3, date of approval July 10, 2020). All children provided their verbal consent to participate in the study and were informed about their right to withdraw from the study at any time during the testing. Written informed consent was also obtained from a parent/guardian of each child.

## Results

### Data Labeling Aggregation

First, we explored the level of consistency between two annotators for various IC classes. Due to limited available data and only two annotators, we decided to merge some classes with a small number of label matches between annotators. One reason for this small number could be the possible difference in labeling strategies between the experts, as was discussed in the section “Materials and Methods.” The final manipulations with class labels are:

• Eyes, Horizontal eye movement, Vertical eye movement were merged to the one Eye movement class.
• Line noise labels were dropped due to a lack of actual line noise in available data.
• Alpha and mu labels were checked to be marked as a Brain label too.

For the rest of the IC classes, we used the following aggregation strategies based on each class’s total number of positive samples (see Table 1). When the samples of a particular class were poorly represented, we took *Majority vote strategy* to have enough labeled samples for the model fitting; otherwise, we took Probabilistic vote strategy. The details of *Majority vote* and Probabilistic vote are explained in the section “Materials and Methods.”

**TABLE 1:** IC classes and corresponding aggregation strategies based on the total number of positive samples of each class.

**IC class**	**Brain activity**	**Alpha brain activity**	**Mu brain activity**	**Eyes**	**Muscles**	**Heart**	**Channel noise**
**Strategy**	2	1	1	2	2	1	2

The final number of positive labels and concordance between the two experts are shown in Table 2.

**TABLE 2:** Number of samples and Cohen’s kappa for each class.

**Label**	**Number of samples**	**Cohen’s kappa**
Brain	449	0.47
Alpha	60	0.13
Mu	92	0.22
Eyes	78	0.10
Muscles	135	0.36
Heart	231	0.04
Channel noise	48	0.12

According to arbitrary settled thresholds (Landis and Koch, 1977), the agreement between two experts’ opinions was highest but still moderate (<0.4) only for labeling the ICs of brain signals. The other ICs were labeled with a relatively poor agreement between experts (Table 2). The Inter-expert correlation between our experts equals 0.43, and the approximate level of agreement was also reviewed (Pion-Tonachini et al., 2019). Based on the experts’ comments, we understood that many IC components contain more than one activity type. This mixture led to uncertainty for experts’ labeling strategy. Summing up their annotations and based on the comments, we can conclude that one expert was inclined to label only those components where a clear pattern of chosen IC class could be detected. Another expert labeled all activity types present at given components, even when there was only a slight indication of its presence in multi-nature ICs. This difference in labeling strategies produced relatively poor agreement even for (usually well recognized) Eyes activity. The annotation dataset is available via http://alice.adase.org/downloads.

### Independent Component Classification

**FIGURE 6:**
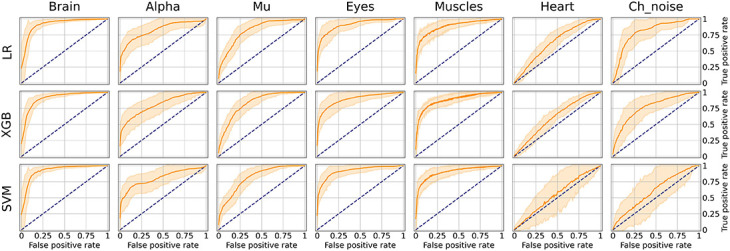
Aggregated Receiver Operating Characteristic (ROC) curves for all IC types and ML models. The solid line indicates the mean curve and the colored area indicates the 95% confidence interval for the ROC curve. The best classification results were achieved for the Brain Muscles and Eyes ICs. For the Alpha Mu and Channel Noise classes, the scores are also high, however, the stability is lower, especially in the case of detecting Channel Noise using SVM. Finally, the performance on Heart components was poor, which could be due to low expert concordance. The blue line on each plot represents the no-skill classifier which assigns labels at random. Thus, we can consider the performance of a particular model on a particular label type statistically significant, if the confidence interval lies above the blue line. Thus, most of our models classify the components significantly better than at chance, expert for SVM that was not able to do it for Heart and Channel noise component.

As it can be seen from Table 2, many classes are relatively small. This leads to imbalanced classification tasks, for example, for Alpha, Mu, and Channel noise IC classes. In this case, Precision-Recall (PR) curve better reflects classifier performance compared to the conventional ROC-AUC curve. So, we explored LR, XGB, and SVM as ML models and calculated both ROC-AUC and PR-AUC scores as performance measures. We selected among three models for each IC type separately. All the models showed comparable performance for most ICs classes (see Figure 6 for ROC curves and Table 3 for values) based on ROC-AUC curves. Brain, Eyes, and Muscles models showed the best performance among others with ROC-AUC greater than 0.9. We could not train a good model for Heart ICs detection due to inadequate labeling as suggested by the lack of consistency among experts and probably not specific extracted features.

**TABLE 3:** Average ROC-AUC values and their standard deviations.

**ROC-AUC**	**Brain**	**Alpha**	**Mu**	**Eyes**	**Muscles**	**Heart**	**Channel noise**
Logistic regression	0.93 (±0.02)	0.83 (±0.05)	0.83 (±0.03)	0.91 (±0.03)	0.89 (±0.03)	0.64 (±0.04)	0.81 (±0.05)
XGBoost	0.92 (±0.02)	0.81 (±0.05)	0.83 (±0.03)	0.89 (±0.04)	0.88 (±0.02)	0.61 (±0.03)	0.77 (±0.05)
Support vector machine	0.93 (±0.02)	0.81 (±0.05)	0.82 (±0.03)	0.92 (±0.03)	0.90 (±0.03)	0.55 (±0.10)	0.61 (±0.09)

*Mean ± St. deviation.*

**FIGURE 7:**
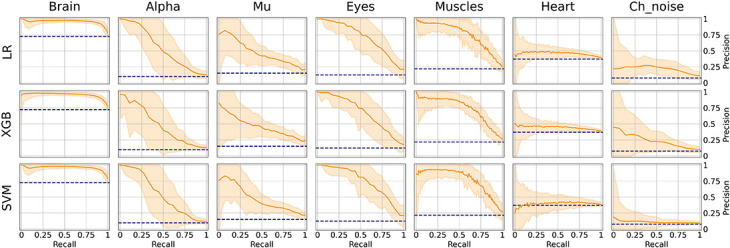
Aggregated Precision Recall (PR) curves for all IC types and ML models. The solid line indicates the mean curve and the colored area indicates the 95% confidence interval for the PR curve. PR curves better indicate classification performance in case of imbalanced data, which can be seen in worse results for Alpha, Mu, Eyes, and especially Channel Noise IC types, all of which have fewer positive labels compared to Brain or Muscles IC classes. As with the ROC curves, we can claim that on all IC types except for Heart and Channel Noise, our models perform significantly better than the unskilled classifier.

However, the picture was different when analyzing PR curves and PR-AUC values (see Figure 7 and Table 4). As we mentioned, PR curves better indicate classification performance in case of imbalanced data, which results in worse performance for Alpha, Mu, and Eyes IC types, all of which have fewer positive labels than Brain or Muscles IC classes. It also can be seen that for Heart and Channel Noise classes, all of the models and SVM in particular performed poorly. The possible reasons for this might be both a small number of samples in each class and a low level of agreement between the annotators resulting in poor labeling quality and lack of robustness. Probably, new robust predictive features should be developed to address these types of artifacts. We also provided F1-score values (see Table 5), alternative statistics based on precision-recall interaction. The need for further investigation of the models’ performance on Heart and Channel Noise IC classes is also backed up by the low F1-score, which is significantly lower than the rest IC types.

**TABLE 4:** Average PR-AUC values and their standard deviations.

**PR-AUC**	**Brain**	**Alpha**	**Mu**	**Eyes**	**Muscles**	**Heart**	**Channel noise**
Logistic regression	0.96 (±0.02)	0.59 (±0.08)	0.50 (±0.07)	0.74 (±0.06)	0.77 (±0.05)	0.46 (±0.04)	0.23 (±0.06)
XGBoost	0.96 (±0.01)	0.54 (±0.10)	0.48 (±0.07)	0.71 (±0.07)	0.75 (±0.05)	0.45 (±0.03)	0.27 (±0.09)
Support vector machine	0.96 (±0.02)	0.59 (±0.08)	0.49 (±0.07)	0.76 (±0.06)	0.79 (±0.05)	0.41 (±0.07)	0.13 (±0.04)

*Mean ± St. deviation.*

**TABLE 5:** Average F1-scores and their standard deviations.

**PR-AUC**	**Brain**	**Alpha**	**Mu**	**Eyes**	**Muscles**	**Heart**	**Channel noise**
Logistic regression	0.92 (±0.01)	0.50 (±0.11)	0.31 (±0.08)	0.62 (±0.08)	0.66 (±0.05)	0.14 (±0.05)	0.00 (±0.00)
XGBoost	0.91 (±0.01)	0.50 (±0.12)	0.39 (±0.08)	0.64 (±0.07)	0.69 (±0.04)	0.40 (±0.04)	0.18 (±0.11)
Support vector machine	0.92 (±0.01)	0.42 (±0.10)	0.20 (±0.09)	0.63 (±0.07)	0.72 (±0.04)	0.01 (±0.02)	0.00 (±0.00)

*Mean ± St. deviation.*

It is worth mentioning that the main reason for measuring PR-AUC was to compare the performance of the models with each other. In general, specific PR-AUC values, unlike ROC-AUC, do not reflect the model’s performance. For that, it is better to refer to the PR curve itself. Each point on this curve corresponds to certain precision and recall levels closely related to type I and II errors, respectively. We could achieve this by choosing the appropriate threshold (by default, each model predicts probabilities for each class that can be interpreted as either True or False by comparing with the threshold value). To better illustrate this idea, we suggest the following example. Supposing, we want to detect muscles with the recall of 0.75 (that is, we will detect 75% ICs with muscular activities). Then, by looking at Figure 7, we can see that SVM will achieve a precision value of about 0.7, which means that out of all ICs selected, about 70% will correspond to Muscles.

We chose an ML model for each IC type based on the ROC-AUC score if the class is relatively balanced (Brain and Heart and Muscles) and based on PR-AUC if the class is unbalanced. Thus, we selected PR for Brain, Alpha Mu, and Heart, XGB for Channel Noise, and SVM for Eyes and Muscles.

### Additional Tests

**FIGURE 8:**
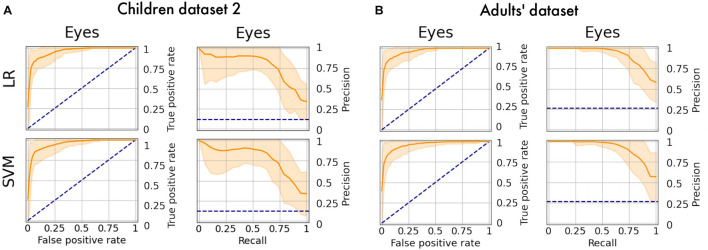
PR curves and ROC curves for Eyes class for additional datasets. The performance rate for additional datasets illustrated using ROC-curve and PR-curve. The solid line indicates the mean curve and the colored area indicates the 95% confidence interval for the curves. **(A)** Is a pair of plots for Children dataset 2 and we observe that both plots illustrate high quality of predictions for Eye components. **(B)** Is a pair of plots that show a high performance rate for Eye components for Adults’ dataset.

The obtained models were applied to additional datasets to decode eye artifacts. The model trained on the main dataset showed a controversial result while being tested on Children dataset 2 (F1-score = 0.12; PR-AUC = 0.18; ROC-AUC = 0.5). Nonetheless, the models perform well after retraining (Figure 8A) with PR-AUC values on the level of 0.94 (see Table 6). The latter implies that model re-training is beneficial for new datasets (even of the same age cohort) making model flexibility an important part of the proposed framework. After aggregation of experts labeling the dataset consisted of 64 eye components out of 527.

**TABLE 6:** Performance rate on additional datasets.

	**Children dataset 2**			**Adults’ dataset**		
	**ROC-AUC**	**PR-AUC**	**F1-score**	**ROC-AUC**	**PR-AUC**	**F1-score**
Logistic regression	0.96	0.81	0.76	0.97	0.94	0.83
Support vector machine	0.96	0.81	0.76	0.97	0.94	0.83

We also tested model performance on eye components from Adults’ dataset (with 61 labeled eye-components out of 337). The model trained on initial dataset again performed weakly (F1 = 0.36; ROC-AUC = 0.61; PR-AUC = 0.65), while the re-trained models showed a dramatic increase in all quality measures (see Figure 8B). The obtained performance rate makes up 0.95 for PR-AUC. Thus, we examined that ALICE machine learning pipeline is also appropriate for adult EEG.

## Discussion

Independent component analysis is a powerful tool for the segregation of various types of activities from the raw EEG data. It is widely used for the detection of different artifacts such as eye blinks or muscle contractions. Nevertheless, IC signals’ correspondence to any class of activity largely depends on a particular expert, affecting the study results. This issue is worth highlighting as the application of ICA in EEG studies becomes more and more popular. The ALICE toolbox is a particular instrument to resolve these issues.

The developed web application stores ICA data and makes it publicly available. This data includes IC annotations given by experts, which assign each component to the appropriate category. Moreover, the annotated dataset expands using the interface where each expert can make their labeling. ALICE’s goal is to build a community where experts from neuroscience, neurophysiology, and other related areas, share their ICA data and encourage each other to make the annotations. Our study’s low Cohen’s kappa coefficient and low inter-expert correlation in IC annotation point to high disagreement in components annotation evident even between two experts. Noteworthy, the only other crowdsourcing platform for IC classification [ICLabel, (Pion-Tonachini et al., 2019)] also report similar results: their mean inter-expert correlation was 0.50, ranging from 0.46 to 0.65, clearly pointing to different strategies of identification ICs. This finding emphasizes the need to study the reason for such low agreement between experts and to develop an automatic IC classification toolbox that will work objectively.

The ALICE has the potential to unite the efforts of experts from different fields that are vital to developing an ML model that could be used in EEG studies for the objective assessment of various artifacts. Our baseline model is clear evidence that ICA artifacts selection can be easily automated using ML approaches. The novel aspects of the work include the algorithm for mu and alpha rhythm detection. The critical point is that the model is publicly available and additionally can be used as a pre-trained model for posterior modifications for other tasks.

Subjective labeling and ML training was performed on a dataset of ICs obtained on EEG data recorded in pre-school and school-age children, a population with usually many artifacts. This type of dataset is relatively unrepresented in the previous research on automatic IC extraction. The main work with infant ICA was done by Adjusted ADJUST algorithm (Leach et al., 2020) does not rely on machine learning techniques. The dataset consists of 630 ICA components acquired from 20 children, making up a unique publicly available dataset that can be used for various goals, e.g., for refitting new private models for ICA detection.

There are several points for future development of the project related to the annotation module and the ML module. The annotation module advances are related to the reorganization of available classes to mark into a hierarchical structure. Users can first select the artifact and specify it more precisely, for example, Eyes->Horizontal eye movements. Moreover, the first trial of expert annotations forces us to reestablish an expert policy and force them to choose no more than two IC classes to train our models using representative samples.

The ML module showed a high-performance rate for most classes. Although the Heart class was not detected, the reason for that is the lack of class representatives and a low agreement between the annotators. Moreover, the Mu/Alpha rhythms and Eyes results were also obtained with fewer data samples. Nevertheless, the ALICE approach (including newly designed features for Mu and Alpha classes) showed good classification accuracy for ICs labeling even though the agreement between expert opinions was relatively poor. Still, for Heart and Channel Noise classes, none of the trained models worked well. Probably new robust predictive features or more complex ML models (i.e., based on convolutional neural networks) should also be developed to address these types of artifacts. We compared the performance of our algorithms with results reported in other studies. In Pion-Tonachini et al. (2019) authors report ROC curves with F1 scores. Eyes class F1 score is greater than 0.9, brain and muscles classes are in the range between 0.8 and 0.9, which is higher than results obtained using our model; at the same time, the heart class, like in our case, is reported as uninformative. In Tamburro et al. (2018), the authors reported accuracy, sensitivity, and false omission rates and provided complete data for eye movements, eye blinks, and muscle activity. The resulted F1 scores were greater than 0.9. In terms of our model, the low agreement between experts as an outcome of different labeling approaches might affect the final score.

Nevertheless, with additional datasets we discovered that the result can gain higher values for Eyes IC class with F1 score on the level of 0.87. Such values can be achieved for both adult EEG as well as for children EEG. This result implies that ALICE ML pipeline is robust to datasets of different ages. On the other hand, models require retraining to be suitable for data of different age or data of different ICA algorithm. This observation examined that database requires more components to show stable result over any type of dataset.

The current performance of ML algorithms in the ALICE toolbox is based mainly on two experts’ estimations, whereas a manifold of professional annotations produces more objective estimates for components labeling. In future research, we aim to invite the wider expert community to label their datasets and expand current models’ abilities or future models to define the functional nature of IC components. Thus, we encourage any reader to become a part of the ALICE project. More information about the potential contribution is provided on our web site http://alice.adase.org/docs/contribute.

To summarize, the main improvements implemented in ALICE as compared to previously developed toolboxes are the following:

• The ALICE toolbox allows not only detection of noisy IC but also automatic identifications of components related to the functional brain oscillations such as alpha and mu-rhythm.
• The ALICE project accumulates different benchmarks based on crowdsourced visual labeling of ICs collected from publicly available and in-house EEG recordings, resulting in a constantly growing high-quality IC dataset.
• ALICE implements the new strategy for consentient labeling of ICs by several experts.
• ALICE allows supervised ML model training and re-training on available data subsamples for better performance in specific tasks (i.e., movement artifact detection in healthy or autistic children).
• Finally, ALICE provides a platform for EEG artifact detection model comparison as well as a platform for neuroscientist self-assessment based on established performance metrics.

Thus, strength of the ALICE project implies the creation and constant updating of the IC database, which will be used for continuous improvement of ML algorithms for automatic labeling and extraction of non-brain signals from EEG. The developed toolbox will be available to the scientific community in an online service and open-source codes.

## Data Availability Statement

The datasets presented in this study can be found in online repositories. The names of the repository/repositories and accession number(s) can be found in the article/supplementary material.

## Ethics Statement

The studies involving human participants were reviewed and approved by the Ethics Committee of the Institute of Higher Nervous Activity and Neurophysiology or Russian Academy of Sciences. Written informed consent to participate in this study was provided by adult volunteers or the children participants’ legal guardian/next of kin.

## Author Contributions

GS, AL, MN, OM, IP, GP, AR, OS, and MS conceived and designed the study. AL and GS developed the tool. AL, MN, and GS developed the model. OS, GS, and AR provided dataset. GP and AR analyzed the ICA data. OS, OM, MS, GS, AL, and MN wrote the manuscript. All authors revised the article.

## Conflict of Interest

The authors declare that the research was conducted in the absence of any commercial or financial relationships that could be construed as a potential conflict of interest.

## Publisher’s Note

All claims expressed in this article are solely those of the authors and do not necessarily represent those of their affiliated organizations, or those of the publisher, the editors and the reviewers. Any product that may be evaluated in this article, or claim that may be made by its manufacturer, is not guaranteed or endorsed by the publisher.
